# Machine learning-enabled systematic review on coded healthcare data in heart failure research

**DOI:** 10.1093/ehjdh/ztaf123

**Published:** 2025-10-23

**Authors:** Asgher Champsi, Karin T Slater, Simrat Gill, Tomasz Dyszynski, Megan Schröder, Kiliana Suzart-Woischnik, Benoit Tyl, Guillaume Allée, Alfonso Sartorius, R Thomas Lumbers, Folkert W Asselbergs, Diederick E Grobbee, Georgios Gkoutos, Dipak Kotecha

**Affiliations:** Department of Cardiovascular Sciences, University of Birmingham, Medical School, Vincent Drive, Birmingham B15 2TT, UK; NIHR Birmingham Biomedical Research Centre, University Hospitals Birmingham NHS Foundation Trust, Birmingham B15 2GW, UK; Centre for Health Data Science, University of Birmingham, Birmingham B15 2TT, UK; Institute of Cancer and Genomics, University of Birmingham, Birmingham B15 2TT, UK; Department of Cardiovascular Sciences, University of Birmingham, Medical School, Vincent Drive, Birmingham B15 2TT, UK; Bayer AG, Berlin, Germany; Boehringer Ingelheim, Ingelheim, Germany; Bayer AG, Berlin, Germany; Bayer Healthcare SAS, La Garenne-Colombes, France; Servier Laboratories, Paris, France; Servier Laboratories, Madrid, Spain; Institute of Health Informatics, University College London, London, UK; Institute of Health Informatics, University College London, London, UK; The National Institute for Health and Care Research (NIHR) University College London Biomedical Research Centre (BRC), University College London, London NW1 2PG, UK; Department of Cardiology, Amsterdam University Medical Center, Amsterdam, the Netherlands; Julius Center, University Medical Center Utrecht, Universiteitsweg 100, 3584 CG Utrecht, the Netherlands; Centre for Health Data Science, University of Birmingham, Birmingham B15 2TT, UK; Institute of Cancer and Genomics, University of Birmingham, Birmingham B15 2TT, UK; Department of Cardiovascular Sciences, University of Birmingham, Medical School, Vincent Drive, Birmingham B15 2TT, UK; Julius Center, University Medical Center Utrecht, Universiteitsweg 100, 3584 CG Utrecht, the Netherlands; NIHR Birmingham Biomedical Research Centre, University Hospitals Birmingham NHS Foundation Trust, Birmingham B15 2GW, UK

**Keywords:** Heart failure, Coding, Research, Transparency, Methodology

## Abstract

**Aims:**

Coded healthcare data are now commonly used in clinical research. This study aimed to assess the transparency of reporting within heart failure studies and employ machine learning to facilitate larger-scale evaluation.

**Methods & Results:**

A systematic search of EMBASE and MEDLINE (2015–2020) identified 4279 heart failure studies with accessible Extensible Markup Language published in the top 25 journals by impact factor. Manual extraction in a random sample of 170 studies by independent human reviewers characterized 40 studies (23.5%) that used coded healthcare data, with 34 of these (85%) reporting doing so and only 19 (47.5%) providing clear descriptions of dataset construction and linkage. Another 420 studies underwent manual annotation to further train a Natural Language Processing (NLP) model designed for this study to automate and upscale review. The NLP model processed 3689 studies with a high level of internal accuracy (area under the receiver operating characteristic curve 0.97 and F1 score 0.96). Overall, the NLP approach identified 782 studies (21.2%) that reported coded healthcare data usage (95% CI 19.8–20.9%). No correlation was found between the reporting of coded healthcare data use and the publication year (r = ^−^0.05; *P* = 0.21) or citation count (r = ^−^0.13; *P* = 0.12).

**Conclusion:**

One-fifth of contemporary heart failure research articles are already reporting the use of coded healthcare data, with at-scale evaluation facilitated by a machine-learning model. The limited transparency on how coded healthcare data were used in studies highlights the need for quality standards such as the CODE-EHR framework for the use of healthcare data in research.

## Graphical Abstract

**Graphical Abstract:**
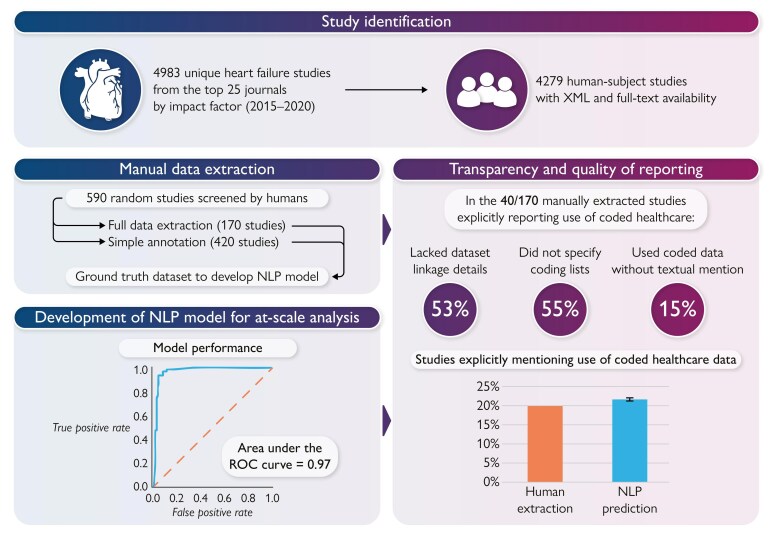
Visual abstract describing the study design and primary results. ROC = Receiver operating characteristic; NLP = Natural language processing; XML = Extensible Markup Language.

## Background

The use of large-scale data from routine clinical care has the potential to expand our understanding of disease and develop more effective prevention and treatment strategies. Health data science has undergone rapid development in recent years and electronic healthcare record (EHR) systems are now in widespread use, enabling clinical episodes to be summarized using standardized, coded data elements. Although historically this practice primarily supported administrative and billing purposes, contemporary literature underscores its increasing utility for clinical research.^1^ Structured labels facilitate large-scale population studies and real-world evidence generation, although limitations must be recognized such as incomplete data, coding inaccuracies, and variability across institutions and regions. In addition, there is a clear lack of transparency about how EHR data are used and how medical conditions and outcomes are defined in clinical research, undermining the value of scientific findings and limiting external validation. Addressing these issues was the rationale for the global CODE-EHR multi-stakeholder framework on research using health data.^2^

Using heart failure as an exemplar, the purpose of this study was to evaluate transparency in the reporting of coded healthcare data. We conducted a systematic search and manually curated a sample of studies to evaluate reporting practices. The extracted findings were then used to train a machine learning algorithm using Natural Language Processing (NLP). We hypothesized that reporting of the use of healthcare data in research studies was suboptimal, and NLP would enable automated and scalable analysis across a volume of journal articles too large for manual review to address the transparency of reporting.

## Methods

This programme of work was initiated under the European Union (EU) Innovative Medicines Initiative BigData@Heart, involving collaboration between academic and industry partners. This systematic review has been reported in accordance with PRISMA recommendations, and the protocol was prospectively published on the EU Open Research Repository.^3^ As many of the included studies used EHR data, the CODE-EHR framework^2^ was applied, with this study meeting all five of the minimum standards, and two out of five domains meeting preferred criteria; see Supplementary material online, *Table S1*.

### Search strategy and eligibility criteria

EMBASE and MEDLINE databases were systematically searched, spanning the period from 1st January 2015 to 31st December 2020. A broad search description for heart failure was used to identify relevant studies published in the top 25 journals based on impact factor rating from the Clarivate Analytics 2019 categories of ‘Cardiac & Cardiovascular Systems’ or ‘Medicine, General & Internal’; see Supplementary material online, *Table S2* for search criteria and Supplementary material online, *Table S3* for the full list of journals. Studies were included if full text was available and Extensible Markup Language (XML) data could be extracted for the purposes of NLP. Reviews, case reports, basic science research, animal studies, and non-English articles were excluded.

### Data extraction

Manual data extraction was performed on 180 studies that were randomly selected using a computer-generated random number sequence. Each study was independently assessed by two reviewers using a standardized data extraction form, with ten studies excluded as they did not involve human participants. Both the main publication and the supplementary materials were reviewed. Extracted parameters included whether the study explicitly mentioned the use of coded healthcare data, details of dataset construction and linkage, pre-specification of coding schemes, and the coding system(s) utilized. Discrepancies were resolved through consensus discussion or adjudication by a third reviewer. The manually extracted dataset characterized the quality and completeness of reporting practices, and was used to develop the NLP model. Following this, a separate set of 420 studies were randomly selected for manual annotation by two human reviewers to determine whether each study explicitly mentioned the use of coded healthcare data or not, with the aim of improving the accuracy of the NLP model. Discrepancies in annotation were resolved through independent adjudication by a third reviewer.

### Model synthesis

For the NLP approach, a text classification pipeline^4–6^ was developed to classify documents as to whether they explicitly reported use of healthcare coding in their methodology. The dataset was further split into a training set and a test set (see Supplementary material online, *Table S4*). The training set was used to develop a rule-based sentence-matching system, composed of 24 regular expression patterns, to identify mentions of healthcare coding. These were added to a set of explicit patterns that matched particular codes for heart failure, such as the International Classification of Diseases (ICD-10) code ‘I50.0’, which were queried from the Health Data Research (HDR) UK Phenotype Library. The entire training set was then evaluated for matches to any of these 323 codes. These codes were used as the basis for feature vectors, which were used to train the model. The manually annotated set of 420 studies were used to enhance the model. The Stanford CoreNLP^7^ was employed to identify mentions of healthcare coding using this system.

To complete evaluation of whether articles explicitly mentioned healthcare coding, a machine learning classification approach was used. The purpose of this was to further resolve ambiguity for documents that matched one of the rules, by creating a classifier to learn which matched rules, or relations between matched rules, indicated actual mentions of healthcare coding, rather than incidental or erroneous mentions. For each document, a vector was created consisting of the Term Frequency—Inverse Document Frequency (TF-IDF) value (to assess the relative importance of a term in each document in the collection of studies) for each of the matched instances of vocabulary patterns across the training set. A stochastic gradient descent classifier was then employed to reinforce the training set; this was trained using the manually marked documents in the training set, a 5-cross-fold grid search for optimization of hyperparameters, and with Synthetic Minority Oversampling Technique (SMOTE) data augmentation.

### Outcomes

The primary outcome was the proportion of heart failure studies that utilized coded healthcare data to define disease or ascertain outcomes. The secondary outcomes included assessment of study characteristics, dataset construction, data linkage and coding schemes, the lists used to define HF, and how clearly and unambiguously the studies reported their use of coded healthcare data. Process outcomes included performance evaluation of the NLP model vs. manual human extraction, including disagreement between the human operators used to evaluate inter-annotator agreement. It was not feasible or necessary to assess the risk of bias for each individual study as this review was focused on study methodology rather than results.

### Statistical analysis

Descriptive statistics were generated to summarize and compare relevant HF study characteristics. Logistic regression was employed to develop a multivariable model predicting the explicit mention of HF coding. A two-tailed *P*-value <0.05 was used to denote statistical significance. Predictors in the model included journal, citation count, and publication year, all retrieved from the CrossRef database on 9 January 2023. Model discrimination was assessed using the concordance statistic (C-statistic), and calibration was evaluated using a calibration plot. Point biserial correlation was used to examine the relationship between the numeric publication year and the outcome. Publication year was also included in the model as a categorical factor to allow for the possibility of capturing non-linear relationships. The analysis was conducted using R and the following packages: Caret, forestplot, pROC, broom, readr, and dplyr. Python, with the habanero library, was utilized to access CrossRef records.

## Results

**Figure 1:**
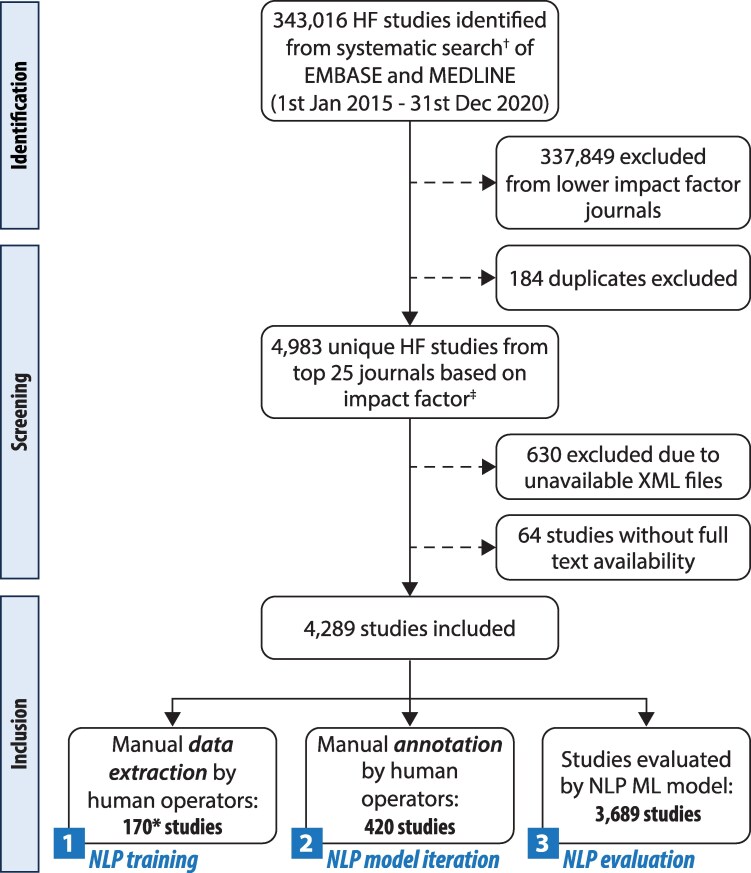
Study flow diagram. Flow chart of the search results and study inclusions. † The search description for heart failure that was used to identify relevant studies can be found in Supplementary material online, *Table S2*. ‡ Impact factor rating was in accordance with Clarivate Analytics 2019 categories ‘Cardiac & Cardiovascular Systems’ and ‘Medicine, General & Internal’. *10 articles excluded as not based on human participants. HF = Heart failure; ML = Machine learning; NLP = Natural language processing; XML = Extensible Markup Language.

A total of 4289 unique studies matched the search criteria, were published in the top 25 journals by impact factor, and had full text and XML availability (*Figure 1*).

### Transparency and quality of reporting

From the 170 random studies that underwent manual data extraction and evaluation by human operators, 118 (69.4%) were observational studies and 33 (19.4%) were randomized controlled trials. The remaining 19 studies (11.2%) included genetic association, validation, and methodology-focused research. There was broad geographical distribution, with the commonest region being Europe (74 studies, 43.5%), and clinical research or registries the most common source of healthcare data; *Table 1*. Evaluation of the article text by human reviewers identified 40 studies (23.5%) that had used coded healthcare data. These studies were a mix of primary and secondary care, were mostly large (32 studies with >1000 participants, 80.0%) and typically used versions of the ICD coding system; *Table 2*. 21/40 studies (52.5%) did not provide clear details on dataset construction or linkage, and 22/40 studies (55.0%) did not have pre-specified or pre-published coding lists. 6/40 studies (15.0%) did not explicitly mention that the research had used coded data in some element of the study, despite evidence of this in the main or Supplementary material. Including the additional manually annotated studies, a total of 119/590 research articles (20.2%) were found to explicitly mention the use of coded healthcare data (see Supplementary material online, *Table S4*).

**Table 1:** Characteristics of studies that underwent manual data extraction

Characteristics of studies (total *n* = 170)	Number of studies (%)
**Study design**	
Observational	118 (69.4%)
Randomized controlled trial	33 (19.4%)
Genetic association study	4 (2.4%)
Validation study of disease definition	2 (1.2%)
Other	13 (7.6%)
**Region of study origin**	
Multiple Regions	25 (14.7%)
Asia-Pacific/Middle East/Africa	26 (15.3%)
Europe	74 (43.5%)
North America	36 (21.2%)
Not specified	9 (5.3%)
**Source of healthcare data**	
Administrative/claims	18 (10.6%)
Electronic healthcare records	13 (7.6%)
Registry	40 (23.5%)
Clinical study	89 (52.4%)
Health surveys	1 (0.6%)
Other	9 (5.3%)
**Type of heart failure**	
Reduced ejection fraction	31 (18.2%)
Mid-range ejection fraction	1 (0.6%)
Preserved ejection fraction	7 (4.1%)
All/any/not specified	131 (77.1%)

A summary of the studies with human participants that were accessible at full-text level.

**Table 2:** Characteristics of studies using coded healthcare data

Studies using coded healthcare data (total *n* = 40)	Number of studies (%)
**Study context**	
Primary care/community	13 (32.5%)
Secondary care/hospital	15 (37.5%)
Both primary and secondary care	12 (30.0%)
**Total number of participants in the study**	
<100	1 (2.5%)
100–1000	6 (15.0%)
>1000–10 000	15 (37.5%)
>10 000	18 (45.0%)
**Information on coded data use**	
Explicit mention of coded healthcare data use	34 (85.0%)
Clear description of dataset construction and data sources linkage	19 (47.5%)
Pre-specification and/or publication of relevant code lists	18 (45.0%)
**Purpose of coding**	
Define diseases or comorbidities	11 (27.5%)
Define outcomes	11 (27.5%)
Define both diseases/comorbidities or outcomes	16 (40.0%)
Unspecified	2 (5.0%)
**Codes used** ^a^	
ICD-9	18 (45.0%)
ICD-10	18 (45.0%)
ICD (version 8 or below, or ICD unspecified)	2 (5.0%)
Other (Read codes, SNOMED CT, region specific codes)	2 (5.0%)
Unspecified	9 (22.5%)
**Information on heart failure**	
Use of coding to define heart failure	30 (75.0%)
Validation of coded heart failure definition^b^	11 (36.7%)
Inclusion of LVEF or NTProBNP in heart failure definition	4 (10.0%)

A summary of the studies where human reviewers had evaluated that coded healthcare data were utilized.

^a^8/40 studies used multiple specified code lists.

^b^In the 30 studies that had used coding to define heart failure.

ICD = International Classification of Diseases; LVEF = Left Ventricular Ejection Fraction; NTProBNP = *n*-terminal prohormone of brain natriuretic peptide; SNOMED CT = Systematized Nomenclature of Medicine Clinical Terms.

### Scalable machine-learning assessment

**Figure 2:**
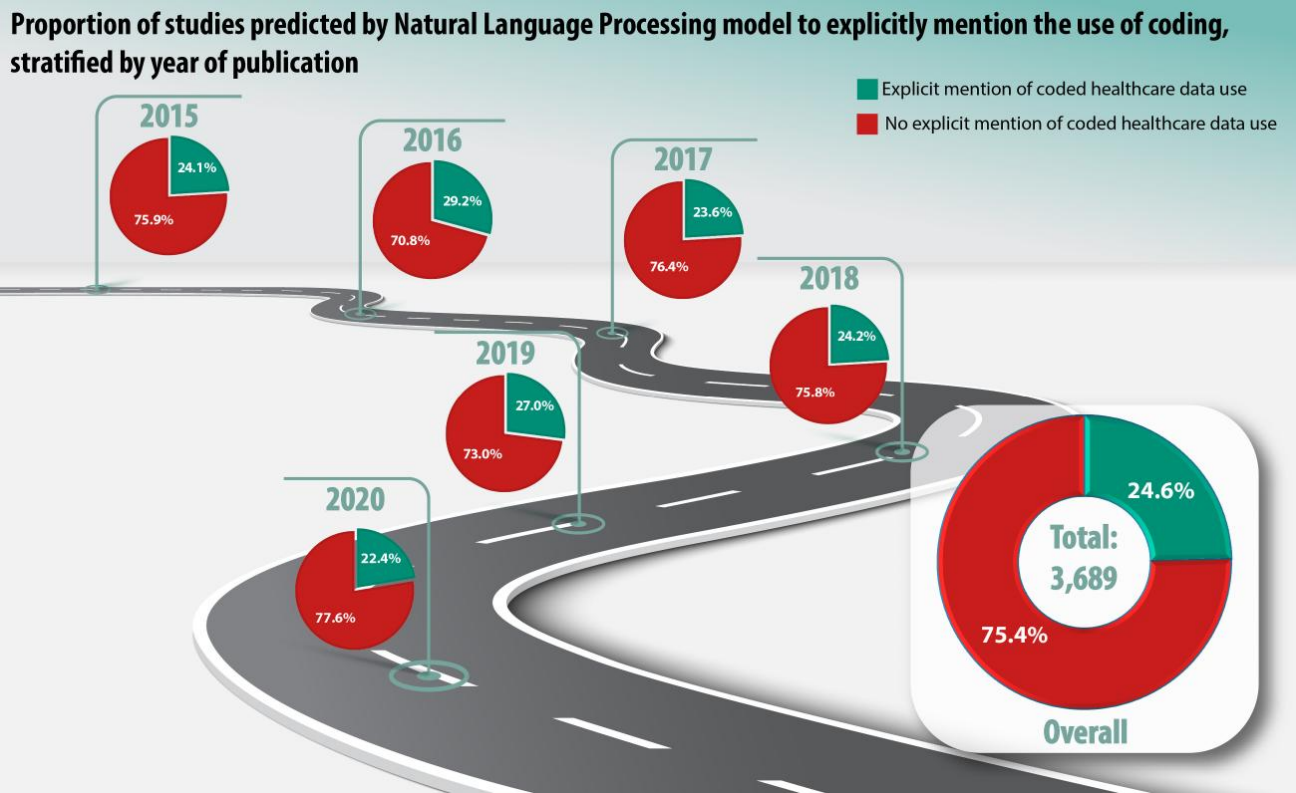
Use of healthcare data coding by year. Pie charts showing the proportions of studies explicitly mentioning the use of coding stratified by year of publication as per the natural language processing model prediction.

Following training and optimization of the model, 3689 unique studies were evaluated by the NLP algorithm, which classified 909 studies (24.6%) as reporting the use of coded healthcare data. Using the precision metric to adjust for the model's precision and based on the manual ‘ground truth’ data, the point estimate of the number of studies predicted by the NLP model for explicitly mentioning the use of coded healthcare data in the wider dataset was 782 (95% CI 761–802), or 21.2% of studies (95% CI 20.6–21.7%). There was no correlation between publication year and the predicted reporting of coded healthcare data usage (correlation coefficient −0.05; *P* = 0.21), with the proportion of studies explicitly reporting coded data remaining stable between 2015 and 2020; *Figure 2*. There was no correlation between citation count and the predicted reporting of coded data use (correlation coefficient −0.13; *P* = 0.12).

### Performance of the NLP model

**Figure 3:**
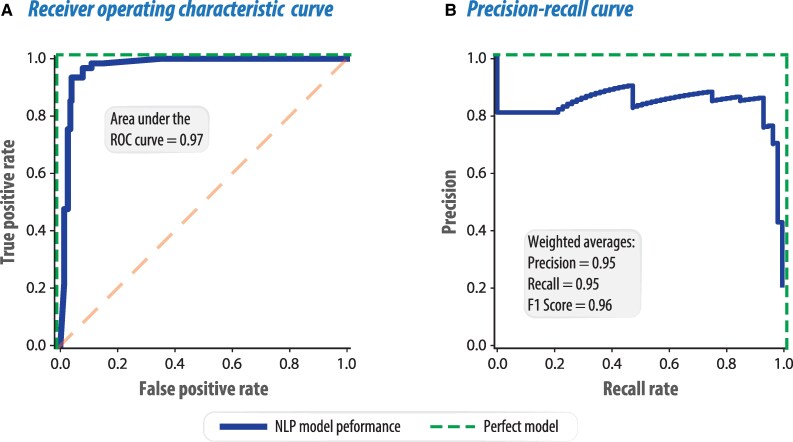
Performance of natural language processing model. Receiver operating characteristic curve (*A*) and precision-recall curve (*B*) for the natural language processing model. ROC = Receiver operating characteristic; NLP = Natural language processing; F1 score = harmonic mean of the precision and recall (max = 1).

The area under the receiver operating characteristic curve on the test set for the NLP model was 0.97; *Figure 3*. The weighted average precision was 0.95 and recall 0.95, with the F1 score indicating accuracy of 0.96 (see Supplementary material online, *Table S5*). The inter-assessor agreement (Cohen's kappa coefficient) was 0.79. The average human-to-machine agreement was 0.78. Introducing a third human adjudicator improved the reliability overall, with an average assessor-to-adjudicator agreement of 0.90 and the human-to-machine agreement rising to 0.87 (*Table 3*).

**Table 3:** Agreement metrics between human reviewers and NLP model

	Pre-adjudication		Post-adjudication by a third human	
Group^a^	Inter-reviewer^b^ agreement	NLP-to-human agreement	Reviewer-adjudicator^b^ Agreement	NLP-to-human agreement
1	0.83	0.78	0.92	0.85
2	0.50	0.72	0.75	0.95
3	0.93	0.83	0.97	0.87
4	0.89	0.84	0.95	0.84
5	0.87	0.78	0.94	0.84
6	0.72	0.74	0.86	0.87
Average	0.79	0.78	0.90	0.87

Agreement represented by Cohen’s kappa coefficient, which ranges from 0 to 1 (with 1 indicating complete agreement).

^a^Groups represent distinct subsets of studies curated by consistent reviewer pairs, enabling assessment of inter-assessor and model agreement across batches. Each group corresponds to a separately reviewed portion of the manually extracted dataset.

^b^Denotes human-to-human agreement.

NLP = Natural language processing.

## Discussion

This study evaluated the transparency of reporting practices related coded healthcare data use in heart failure research, and explored whether NLP could be used to support scalable assessment and tracking of changes in such practices over time. We demonstrate that the reporting of coded healthcare data use is often incomplete or unclear, with fewer than half of the studies describing how datasets were constructed, linked or which coding schemes were used. The NLP machine learning approach confirmed that around one-fifth of contemporary research studies on the topic of heart failure are already using coded healthcare data. This is likely to be an underestimate, as the manual human data extraction identified a number of studies that failed to explicitly mention their use of structured health data sources to define diseases or outcomes. Regardless of the true proportion, our findings highlight the need for more transparency and improvements in research methods, so that clinicians, policymakers and the public can better understand and integrate results, and improve implementation of evidence-based care.

Modern-day EHR systems are gradually becoming the norm, both across developed and developing countries.^8,9^ They have the potential to unlock new insights for observational research and clinical trials,^10,11^ including better representation of the true population at-risk. However, there are considerable challenges in data quality, timeliness and linkage that remain fragmented within and across different healthcare systems.^12^ The CODE-EHR framework was specifically developed as a set of principles and guidelines to enhance the use of structured, routine healthcare data in clinical research.^2^ CODE-EHR was developed by a broad range of stakeholders (regulators, academics, clinicians, patients, payers, journals and industry) and aims for all research using EHR systems to be high quality, transparent and reproducible. The framework uses a set of minimum and preferred standards to systematically embed appropriate dataset construction, data quality, disease and outcome definitions, analysis and governance into the design and execution of such studies.

The human-derived extraction in this study underscores the importance of these data standards. A number of studies failed to mention they had used coded healthcare data, instead inferring this or relegating detail to supplementary documentation. Even in those studies that explicitly acknowledged their use of healthcare data, only a minority adequately discussed data provenance or provided detailed code lists to enable validation. The manual data extraction performed was a labour-intensive process that would not be applicable across a larger volume of studies, hence we developed a more scalable approach using machine learning. Although the depth of understanding is more limited with the NLP method (in this case, distilled to a binary question on reported use of coded healthcare data), it does provide a scalable solution to review published literature en-mass. It also presents an opportunity to address the issue that manually curated reviews include few eligible articles relative to the initial search strategy. Our use of NLP aligns with the broader trend in automation of systematic reviews, where machine learning and NLP have been increasingly used to expedite various steps in the review process, including study searches, screening and data extraction.^13^ This study presents a vanguard application of NLP to enhance systematic reviews through content analysis, extending its use beyond published roles such as extracting the diagnosis and severity of disease,^14^ or supporting clinical quality improvement through abstract screening.^15,16^

The NLP model demonstrated a robust ability to classify documents with high performance, albeit using partitions of the same dataset. Precision and recall metrics were particularly noteworthy for the ‘False’ label, indicating a high accuracy in identifying studies that did not explicitly mention healthcare coding. The model also achieved respectable precision and recall for the ‘True’ label (studies that did report use of coded healthcare data), with slightly lower performance reflecting the inherent complexity of accurately capturing all instances of coded data usage, given the varied ways such usage can be reported in the literature. Although we tend to consider human data extraction as the ‘gold-standard’, there is of course variability across reviewers and challenges in achieving consensus. Inter-reviewer (human-to-human) agreement was substantial, with an average Cohen’s kappa of 0.79, rising to 0.90 after adjudication. This was comparable to inter-reviewer reliability demonstrated in a meta-analysis of 45 studies.^17^ NLP-to-human agreement averaged 0.87 post-adjudication, suggesting high model concordance with human annotation and aligning closely with rates observed in recent published work using large language models.^18^

### Limitations

For feasibility reasons, this study only included articles from a selection of journals. By including the top 25 journals by impact factor, we were able to limit the number of articles where XML were not available that could have biased the NLP model. We also included a very broad range of terms for heart failure, using this condition as an exemplar for cardiovascular disease. Further research would be needed to understand if the findings apply to lower impact journals and other medical conditions. The relatively small size of the training dataset necessitated iteration using the additional human-annotated random study set. As with any model, performance metrics can usually be improved with a more extensive training dataset.^19^ The NLP model performance was internally validated, resulting in high precision and recall metrics, but not externally tested which may limit generalizability. A technical limitation of the approach was that the classifier was built solely on the vector of matching mentions of manually developed patterns (or instances in the text where certain patterns relating to heart failure coding appear). This excludes the potential benefits of vectorizing additional context, such as by creating full sentence or whole document embeddings. Finally, while precision remained very high on the test dataset, there was some reduction in recall, which indicates use of additional terms to explicitly refer to heart failure coding that were not considered in the original set of rule-based patterns. A wider consideration of document content may have bridged this gap in recall. Preliminary explorations using full content embeddings did not yield better results, possibly due to the small training set size. Moving forward, it may be beneficial to explore other advanced machine learning techniques, such as transformer-based models,^13^ to better capture the context around mentions of coded data and leverage richer contextual embeddings that might lead to improved performance. Generative large language models are also developing at a rapid pace.

## Conclusion

One-fifth of contemporary research articles on heart failure report using coded healthcare data, highlighting the critical need for standardized and transparent approaches within clinical research studies based on healthcare records. This study demonstrates a scalable, automated approach to assess transparency in the use of coded healthcare data, with the aim of strengthening reporting standards and clarity in modern research.

## Lead author biography


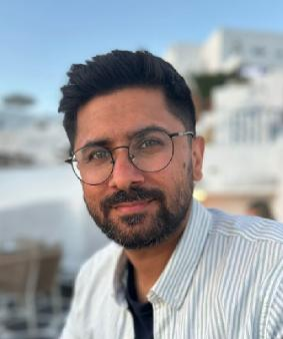


Dr Asgher Champsi is a cardiologist in training and PhD fellow supported by the NIHR Birmingham Biomedical Research Centre. His research focuses on leveraging big data and novel analytic techniques to improve the care of patients with cardiovascular multimorbidity. He is passionate about health data innovation, pragmatic clinical research, addressing health inequalities and improving research participation among underrepresented populations.

## Authors’ contributions

The manual data extraction was conducted independently by all authors. The analysis was conducted by K.T.S., under the direction of D.K. The manuscript was drafted by A.C., K.T.S., and D.K., with all other authors editing the manuscript for intellectual content. D.K. and G.G. provided supervision and were responsible for the decision to submit the manuscript.

Asgher Champsi (MBChB (Data curation [equal]; Formal analysis [supporting]; Writing—original draft [lead]; Writing—review & editing [equal])), Karin T. Slater (PhD (Formal analysis [lead]; Methodology [equal]; Software [lead]; Writing—original draft [supporting])), Simrat Gill (MBChB PhD (Data curation [equal]; Writing—review & editing [equal])), Tomasz Dyszynski (MD (Data curation [equal]; Writing—review & editing [equal])), Megan Schröder (PhD (Data curation [equal]; Formal analysis [supporting]; Writing—review & editing [equal])), Kiliana Suzart-Woischnik (MD MPH (Data curation [equal]; Writing—review & editing [equal])), Benoit Tyl (MD (Data curation [equal]; Writing—review & editing [equal])), Guillaume Allée (PhD (Data curation [equal]; Writing—review & editing [equal])), Alfonso Sartorius (MD (Data curation [equal]; Writing—review & editing [equal])), R. Thomas Lumbers (MBChB PhD (Data curation [equal]; Writing—review & editing [equal])), Folkert W Asselbergs {MD PhD [Writing—review & editing (equal)]}, Diederick E. Grobbee (MD PhD (Conceptualization [supporting]; Writing—review & editing [equal])), Georgios Gkoutos (PhD (Conceptualization [supporting]; Data curation [supporting]; Writing—review & editing [supporting])), and Dipak Kotecha (MBChB PhD (Conceptualization [lead]; Data curation [equal]; Methodology [equal]; Supervision [lead]; Writing—original draft [equal]; Writing—review & editing [equal]))

## Supplementary Material

ztaf123_Supplementary_Data

## Data Availability

Summary data derived from the analysis are available upon reasonable request. Due to the proprietary nature of published studies, sharing individual-level data would require additional permissions from the original publishers.

## Code availability

The machine learning framework for natural language processing and the codes used to train the model are made freely available at: https://github.com/reality/HFCodPaper
