# Supplementary material for: Machine learning-enabled systematic review on coded healthcare data in heart failure research
Source: Eur Heart J Digit Health. 2025 Oct 23;7(1):ztaf123. doi: 10.1093/ehjdh/ztaf123 (PMC12821059; doi:10.1093/ehjdh/ztaf123)
Supplement: ztaf123_Supplementary_Data [file ztaf123_supplementary_data.zip › HF_coding_supplement_21Aug25.docx]

Machine learning-enabled systematic review on coded healthcare data in heart failure research

Supplementary Material

Contents

[Supplementary Table S1: CODE-EHR reporting framework 2](#_Toc197599462)

[Supplementary Table S2: Search strategy description 5](#_Toc197599463)

[Supplementary Table S3: Journal inclusion 6](#_Toc197599464)

[Supplementary Table S4: Sizes of training and test sets, and ground truth definition 7](#_Toc197599465)

[Supplementary Table S5: Precision, recall and F1 scores of the ML model 8](#_Toc197599466)

# Supplementary Table S1: CODE-EHR reporting framework*

| **Domain 1: Dataset construction and linkage** | |
| --- | --- |
| 1. Source of dataset | The dataset for this study was sourced through a systematic search of EMBASE and MEDLINE, focusing on heart failure research articles published between January 2015 and December 2020 in top-impact journals. Human-subject studies with accessible full text and XML data were included for analysis. Data extraction was conducted through manual review by at least two independent human reviews as well as automated through a custom natural language processing (NLP) tool, developed to handle large-scale review and classification. |
| 1. Approach to missing data | Only studies with full-text and extensible markup language (XML) availability were included. Where outcome data was missing within the studies (i.e. where there were gaps in how studies reported coded healthcare data usage), this was categorised separately in the manual extraction and NLP analysis. There was no data imputation performed. |
| 1. Completeness of follow-up | The NLP analysis covered all eligible studies with XML and full-text availability up to the study end date of December 31, 2020. Given this is a review of published studies, completeness in this context refers to the thorough capture and assessment of available publications, rather than longitudinal patient data follow-up. |
| 1. Data linkage | No external data linkage was performed. Each study's data was analysed independently as published, focusing on each article’s specific usage and description of coded healthcare data. |
| **Domain 2: Data fit for purpose** | |
| 1. Origin, process and purpose of data | Data for this analysis originated from published studies in heart failure, where coded healthcare data were often sourced from patient records documented within primary and secondary care settings. The purpose of the data was to aid in the development of an NLP machine learning tool for at-scale evaluation of the transparency of reporting on use of coded healthcare data. |
| 1. Coding systems | Coded data primarily utilised ICD-9, ICD-10, and SNOMED CT. The NLP tool was programmed to recognise these coding systems, ensuring consistent detection of healthcare coding mentions across all included studies. |
| 1. Quality assessment | Only studies from high-impact journals, with accessible XML or full-text data, were included to maintain data quality standards. Quality checks were implemented through manual annotation by human reviewers and a comparison of NLP-generated classifications with manually extracted ground truth data. |
| 1. Potential sources of bias | Potential bias arose from a concentration of studies from high-income regions and limited representation of diverse patient demographics. Nevertheless, disease prevalence and healthcare coding practices align with general UK and EU data, supporting broader applicability of findings within similar healthcare environments. |
| **Domain 3: Disease and outcome definitions** | |
| 1. Definitions | Custom code lists were developed to identify mentions of heart failure conditions, based on ICD, SNOMED and read codes queried from the Health Data Research (HDR) UK Phenotype Library. These codes, numbering 323 in total, were used to train the NLP model. |
| 1. Coding manual | The code lists were structured within the NLP tool, facilitating standardised data extraction for relevant studies. Coding protocols and system details are made available publicly to support research reproducibility. These codes have been made accessible online for transparency: <https://github.com/reality/HFCodPaper/tree/main/data/Vocabulary/PhenotypeCodes> |
| 1. Phenotyping approaches | A rule-based sentence-matching system was developed using the training set, consisting of 24 regular expression patterns to identify mentions of healthcare coding. This system included explicit patterns for specific heart failure codes, such as ICD-10 code 'I50.0', drawn from the Health Data Research (HDR) UK Phenotype Library. The entire training set was evaluated for matches against 323 codes, which served as the basis for feature vectors used to train the NLP model. This approach allowed for consistent identification and classification of studies explicitly reporting heart failure-related coded data. |
| 1. Validation of coding | Validation of the coded lists was performed through comparison with other similar lists on HDR UK Phenotype Library, and iterative NLP model refinement based on annotated training data. |
| **Domain 4: Analysis** | |
| 1. Statistical methods | Refer to the main Methods section for a detailed breakdown of the statistical techniques used in this study. Model performance was validated through standard precision, recall, and F1 score metrics. |
| 1. Machine code | Custom machine learning NLP algorithms were employed to systematically review, classify, and assess the use of coded healthcare data across the included studies. |
| 1. Internal validation | Manual data extraction, alongside cross-validation using human-annotated datasets, ensured model accuracy. |
| 1. External validation | No external validation was performed. |
| **Domain 5: Ethics and governance** | |
| 1. Consent | This study relied solely on published, anonymised research data, with all extracted information sourced from publicly available academic journals. As a secondary analysis of pre-existing studies, no individual patient consent was required. |
| 1. Data privacy | Data privacy was ensured, as only de-identified, published research data were included. No patient-specific information was processed, and privacy standards were upheld throughout the NLP processing pipeline. |
| 1. Patient and public involvement (PPI) | This study benefitted from input and support from the PPI team associated with University Hospitals Birmingham NHS Foundation Trust *cardAIc* initiative (application of Artificial Intelligence to routine healthcare data to benefit patients with cardiovascular disease). The PPI team advised on patient context and ensured the project aligns with broader patient advocacy by promoting transparency and consistency in the use of coded healthcare data for research. |
| 1. Data sharing | Summary data derived from the analysis are available upon reasonable request. Due to the proprietary nature of published studies, sharing individual-level data would require additional permissions from the original publishers. |

*The CODE-EHR framework is designed to improve the transparency, quality, and consistency of research using structured healthcare data by providing minimum and preferred reporting standards across key domains.

A typical use case is presented here:
Mobley, A.R., Subramanian, A., Champsi, A. *et al.* Thromboembolic events and vascular dementia in patients with atrial fibrillation and low apparent stroke risk. *Nat Med* **30**, 2288–2294 (2024). <https://doi.org/10.1038/s41591-024-03049-9>

# Supplementary Table S2: Search strategy description

EMBASE and MEDLINE databases were searched, spanning the period from 1st January 2015 to 31st December 2020. A broad search description for heart failure was used to identify relevant studies:

| **Search term** | **Field** | **Operator** |
| --- | --- | --- |
| Heart failure | Abstract, Title | - |
| Acute heart failure | Abstract, Title  Major term | OR |
| Congestive heart failure | Abstract, Title  Major term | OR |
| Heart ventricle failure | Abstract, Title Major term | OR |
| Cardiopulmonary insufficiency | Abstract, Title Major term | OR |
| Systolic dysfunction | Abstract, Title Major term | OR |
| Diastolic dysfunction | Abstract, Title Major term | OR |

# Supplementary Table S3: Journal inclusion

The top 25 journals were included based on impact factor rating from the Clarivate Analytics 2019 categories of 'Cardiac & Cardiovascular Systems' or 'Medicine, General & Internal':

| **Journal name** |
| --- |
| American Journal of Hypertension |
| Cardiovascular Diabetology |
| Cardiovascular Drugs and Therapy |
| Clinical Journal of the American Society of Nephrology |
| Clinical Research in Cardiology |
| Diabetes Care |
| Disease Markers |
| EP Europace |
| ESC Heart Failure |
| European Heart Journal |
| EHJ Acute cardiovascular care |
| European Journal of Clinical Investigation |
| European Journal of Heart Failure |
| European Journal of Preventive Cardiology |
| Heart |
| Journal of the American Medical Association |
| JAMA Cardiology |
| JAMA Internal Medicine |
| JAMA Network Open |
| Journal of Cardiovascular Translational Research |
| Journal of Clinical Hypertension |
| Journal of Hypertension |
| Journal of the American Heart Association |
| Open Heart |
| PLoS ONE |

# Supplementary Table S4: Sizes of training and test sets, and ground truth definition

| Set | Number of studies, n | Manually curated studies  (composing ‘ground truth’) | | |
| --- | --- | --- | --- | --- |
|  |  | **Total, n** | **True, n (%)** | **False, n (%)** |
| Training | 2491 | 304 | 58 (19.1) | 246 (80.9) |
| Test | 1798 | 286 | 61 (21.3) | 225 (78.7) |
| Total | 4289 | 590 | 119 (20.2) | 471 (79.8) |

This table summarises the distribution of studies used in the supervised machine learning pipeline. A total of 590 studies were manually annotated and used as the ground truth for binary classification of reporting transparency - i.e., whether a study explicitly reported use of coded healthcare data (‘True’) or not (‘False’). These 590 studies were split into a training set (n=304) to develop the classification model and a test set (n=286) for internal performance evaluation.

# Supplementary Table S5: Precision, recall and F1 scores of the ML model

| **Reference to Coded Data** | **Precision** | **Recall** | **F1 score** |
| --- | --- | --- | --- |
| False | 0.98 | 0.96 | 0.97 |
| True | 0.86 | 0.92 | 0.89 |
| Weighted Average | 0.95 | 0.95 | 0.96 |

F1 score = metric combining precision and recall that ranges from 0 to 1, with 1 indicating

perfect accuracy for classification.
